# Consumption of alcohol, cigarettes and illegal substances among physicians and medical students in Brandenburg and Saxony (Germany)

**DOI:** 10.1186/1472-6963-9-219

**Published:** 2009-12-03

**Authors:** Karen Voigt, Sabine Twork, Dirk Mittag, Anne Göbel, Roger Voigt, Jörg Klewer, Joachim Kugler, Stefan R Bornstein, Antje Bergmann

**Affiliations:** 1 Department of General Medicine/Medical Clinic III, Dresden Medical School, Dresden University of Technology, Dresden, Germany; 2 Department of Public Health, Dresden Medical School, Dresden University of Technology, Dresden, Germany; 3 Department of Public Health and Health Care Management, University of Applied Sciences Zwickau, Zwickau, Germany; 4 Medical Clinic III, University Hospital Carl Gustav Carus of the Dresden University of Technology, Dresden, Germany

## Abstract

**Background:**

Patients regard health care professionals as role models for leading a healthy lifestyle. Health care professionals' own behaviour and attitudes concerning healthy lifestyle have an influence in counselling patients. The aim of this study was to assess consumption of alcohol, cigarettes and illegal substances among physicians and medical students in two German states: Brandenburg and Saxony.

**Methods:**

Socio-demographic data and individual risk behaviour was collected by an anonymous self-administered questionnaire. Physicians were approached via mail and students were recruited during tutorials or lectures.

**Results:**

41.6% of physicians and 60.9% of medical students responded to the questionnaire; more than 50% of the respondents in both groups were females. The majority of respondents consumed alcohol at least once per week; median daily alcohol consumption ranged from 3.88 g/d (female medical students) to 12.6 g/d (male physicians). A significantly higher percentage of men (p < 0.05) reported hazardous or harmful drinking compared to women. A quarter of all participating physicians and one third of all students indicated unhealthy alcohol-drinking behaviour. The majority of physicians (85.7%) and medical students (78.5%) were non-smokers. Both groups contained significantly more female non-smokers (p < 0.05). Use of illegal substances was considerably lower in physicians (5.1%) than medical students (33.0%). Male students indicated a significantly (p < 0.001) higher level of illegal drug-use compared to female students.

**Conclusion:**

More than one third of the medical students and health care professionals showed problematic alcohol-drinking behaviour. Although the proportion of non-smokers in the investigated sample was higher than in the general population, when compared to the general population, medical students between 18-24 reported higher consumption of illegal substances.

These results indicate that methods for educating and promoting healthy lifestyle, particularly with respect to excessive alcohol consumption, tobacco use and abuse of illegal drugs should be considered.

## Background

Alcohol consumption, smoking and illegal drug use are important health indicators in accordance with the results of European Community Health Indicators project (ECHI) within the European Commissions Health Monitoring Programme [1]. According to the German Report of Drugs and Addiction [2], approximately 16 million German adults (34%) are smokers and more than 9 million German adults (31% male and 16% female) consume inappropriate levels of alcohol. Five percent of German adults reported use of illegal drugs in the previous 12 months [3].

This indicates a need to educate the German population about the potential negative consequences of alcohol, cigarette smoking and abuse of illegal substances. Physicians play an important role in health education and are often expected to serve as role models for a healthy lifestyle [4-6]. Physicians who lead an unhealthy lifestyle themselves might provide their patients with less effective advice on this topic than their more health-conscious colleagues [7,8]. This study attempts to assess the prevalence of cigarette smoking and level of consumption of alcohol and illegal substances among physicians and medical students.

## Methods

Between April 2004 and May 2006 a voluntary survey was conducted by the Department of General Medicine at the Dresden Medical School. Anonymous questionnaires together with a letter of motivation and prepaid envelope were mailed to all private practice physicians (i.e. general practitioners and surgeons) registered at the National Association of Statuary Health Insurance of Brandenburg and Saxony. Medical students in the 1st, 3rd and 5th academic years at the Dresden Medical School (one of the two public Medical Schools in Saxony) were asked to complete similar questionnaires during tutorials or lectures.

### Questionnaire and statistics

The questionnaire was based on several former surveys [9] and collected information on socio-demographic data, health status (e.g. body mass index, subjective experienced individual health status), occupational stress and reported health behaviour (e.g. consumption of alcohol, cigarettes and illegal substances, received vaccinations [10]). Participants were asked the quantity and type of alcohol consumed in litres per average week. To standardize the data for comparison, litres were converted to grams of alcohol consumed per day (g/d) in calculating daily ingestions. Reference values used for these calculations [11] were: 1) wine: 0.125 l of wine was considered as 11 g of alcohol; 2) beer and mixed drinks: 0.33 l of beer and 0.275 l of mixed drinks were considered as 13 g of alcohol, and; 3) spirits: 4 cl was considered as 11 g of alcohol [12].

All participants were asked to describe their current smoking behaviour and the number of cigarettes smoked daily as well as previous consumption of illegal substances. There was no specific definition of illegal substances in the questionnaire. Data was analysed using SPSS 15.0. Descriptive analysis and nonparametric statistics (χ^2^-tests) were performed, and the level of significance was defined at p < 0.05. In comparing different groups, missing values were pairwise deleted.

### Samples

The total number of responses was 940 with 642 registered physicians and 298 medical students participating. The response rate was 41.6% for the physicians and 60.9% for the students.

The majority of medical students were under the age of 30 years, while the majority of physicians were older than 30 years. The proportion of females was higher in both groups (58.8% and 58.4%, respectively, see Table 1). The number of students was evenly distributed among each of the three academic years (33.0%).

**Table 1:** Socio-demographic sample data

	Medical students (Saxony)		Physicians (Saxony and Brandenburg)	
			GP	Surgeon
			1425	117
Total	**489**		**1542**	

			41.8%	40.2%
response rate	**60.9%**		**41.6%**	

			595	47
Sample included in analysis	**298**		**642**	

*Age*				
<= 30 years	**98.3%**		**7.2%**	
> 30 years	**1.7%**		**92.8%**	

*Gender*				
male: female	**41.2%**	** : 58.8%**	**41.6% : **	**58.4%**

## Results

### Alcohol consumption

More than 75% of the participants reported consumption of alcohol one or more times during an average week. The majority of the respondents (82.5% physicians, 62.8% medical students) drank wine every week, whereas 40% in both groups consumed beer. As the questioning only addressed weekly consumption, it was not possible to identify the phenomenon of binge drinking [2].

The average amount of alcohol consumed among the medical students was significantly higher than among physicians (25.77 vs. 10.88 g/d, respectively). A comparison of the median values (50^th ^percentile) in both groups showed that more students than physicians consumed only few or no amounts of alcohol (5.9 vs. 7.54 g/d, respectively). Nevertheless, a sub-group of students consuming high quantities of alcohol was identified (see Table 2).

**Table 2:** Daily alcohol consumption in g/d among medical students and physicians

		Medical students (n = 298)	Physicians (n = 642)
**Mean +/- standard deviation**		30.84 +/- 56.70	11.58 +/- 15.81

**Mode**		0	0

	**25**	0	2.51
Q**uartile**	**50**	5.89	7.54
	**75**	25.45	14.16

The age and the daily amount of alcohol consumed only correlated significantly in the group of medical students (Kendall-Tau-b, r = 0.104/p = 0.05).

24.8% of the male physicians and 36.5% of the male medical students reported higher ingestions than the recommended daily allowance by the German Nutrition Society on maximal alcohol ingestion (MAAI for men: > 20 g/d). Among the female participants, 25.3% of the physicians and 30.4% of the medical students reported higher ingestions of alcohol than the recommended daily allowance (MAAI for women > 10 g/d) [11].

**Figure 1:**
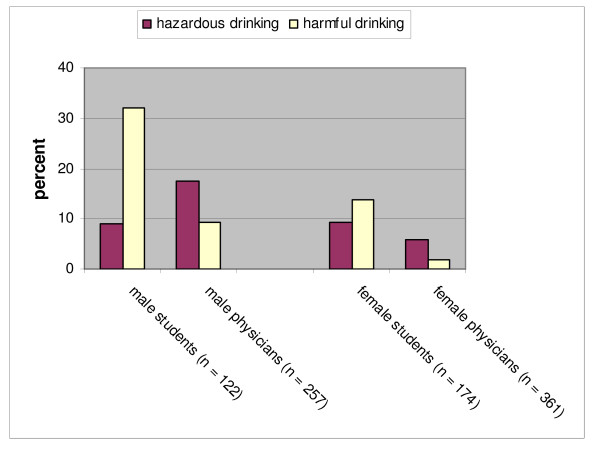
**Proportions of hazardous and harmful drinking among medical students and physicians depending on gender**.

The European Community Health Indicators-Project (ECHI) and the World Health Organisation (WHO) have made distinctions between hazardous and harmful drinking. Hazardous drinking (more than 40 g/d for males and more than 20 g/d for females) is defined as a level of alcohol consumption that may result in physical or psychological damage [13]. Harmful alcohol consumption (more than 60 g/d for males and 40 g/d for females) is defined as a drinking behaviour leading to harming of physical or psychological health [14,15]. According to these reference values, 17.5% of the male physicians and 9% of the male medical students ingested hazardous quantities of alcohol. More than 30% of the male students but less than 10% of the male physicians indicated harmful drinking behaviour (see Figure 1, χ^2 ^= 31.933/p < 0.001).

More female medical students than female physicians consumed significantly hazardous (9.2% vs. 5.8%) and harmful (13.8% vs. 1.9%) quantities of alcohol (χ^2 ^= 33.531/p < 0.001).

### Cigarette smoking

**Figure 2:**
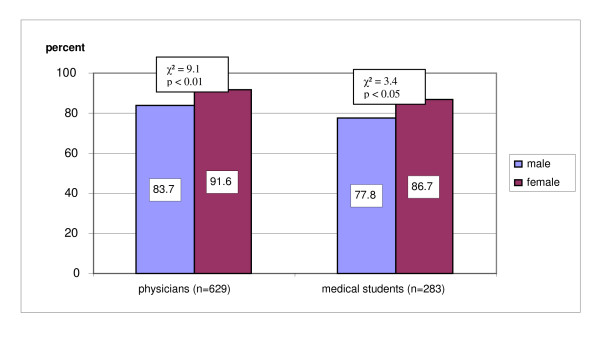
**Proportions of female and male non-smokers among medical students and physicians**.

The majority of physicians (85.7%) and medical students (78.5%) were non-smokers. In both groups fewer females than males reported cigarette smoking (see Figure 2).

No significant correlation between age and non-smoking status was found. No significant gender-related differences in smoking were found between physicians and medical students.

Among smokers (n = 125), 66.4% reported smoking fewer than ten cigarettes per day, while 27.2% reported smoking more than ten but fewer than 20 cigarettes per day. 6.4% of all smokers mentioned a daily smoking rate of more than 20 cigarettes. No relation between age and the number of cigarettes smoked was found.

### Consumption of illegal substances

The majority of physicians (94.9%) and two-thirds of the medical students (66.0%) denied any consumption of illegal substances. Among medical students, fewer female than male students reported consuming illegal substances. Additionally, the one-time or repeated consumption of these substances was significantly lower in female than in male students (χ^2 ^= 24,937/p < 0.001, see Table 3)

**Table 3:** Proportions of students and physicians related to consumption of illegal substances

Amount of consumption	Medical students (n = 291)		Physicians (only Brandenburg, n = 486)	
***Gender***	***male***	***female***	***male***	***female***

**Never**	49.6	77.8	92.5	96.5
**Once**	10.9	7.0	3.3	2.3
**Several times**	39.5	15.2	4.2	1.2

No statistical difference in relation to year of matriculation was found in the group of medical students.

## Discussion

### Sampling and methods

Within the initial distribution group, 61% of the medical students and 42% of the physicians responded to the questionnaire. Among other possibilities, such as method of delivery and time constraints, an intensive political discussion at the time broaching the issues of the survey might have influenced the response behaviour.

The difference in the response rate between the groups may have been affected by the distribution methods (direct distribution during classes vs. distribution by post).

Respondents are proportionally comparable in gender and age to those of physicians in Brandenburg and Saxony [16-18] and of medical students at Dresden Medical School. Therefore, study findings appear to be representative for physicians in private practice and medical students in the eastern states of Germany.

Participation in the study was voluntary and anonymous. It remains unclear whether the response rate was associated with consumption rate of alcohol, cigarettes and illegal substances.

### Alcohol consumption

The proportion of current abstinence in the investigated medical students corresponds to a similar study in medical students from Poland and Germany [19]. Compared to findings related to attitudes towards alcohol drinking behaviour in the European Union, the percentage of physicians reporting abstinence was lower than the average percentages in European males (16%) and females (32%) [20].

Male physicians report consuming significantly higher amounts of alcohol than female physicians, which corresponds to findings in the general German population [11,21]. Furthermore, in comparison to results from a national German health survey [22], the proportion of the investigated male medical students reporting harmful alcohol consumption (20 g/d) was higher than in male Germans 18 to 29 years of age (33% vs. 20%), and the proportion of male physicians reporting harmful alcohol consumption (25%) was lower than in male Germans 30 to 59 years of age (26-32%) respectively. The proportion of investigated female medical students (30.4%) and female physicians (25.3%) reporting harmful consumption of alcohol (> 10 g/d) was remarkably higher than in females in the general German population (7.2% in the group 18-29 years of age, and 15% in the group 30-59 years of age respectively) [22]. Additional German [2,12] and European [20] studies presented findings of harmful alcohol consumption in approximately 25% of the younger population. Consequently, harmful or hazardous consumption of alcohol appears to be a problem in the general German population as well as in health care professionals, especially medical students.

Preventive strategies need to be developed to identify and support health care professionals at risk from high alcohol consumption [12,23]. Moreover, future studies are required to analyse underlying general tendencies, and to determine whether special conditions during medical training (e.g. psychological strains) lead a higher proportion of medical students reporting harmful alcohol consumption. The relationship between alcohol consumption and intake of meals, the time of consumption, and binge drinking should also be studied.

### Smoking

The proportion of cigarette smokers among the investigated group of medical students and physicians is similar to that reported in former studies of health care professionals [24-26]. The percentage in the investigated groups (approximately 25%) is lower than the percentage of smokers in the general European population (European population: 32%; general German population: 30%) [27]. The proportion of non-smokers in this study is higher than in a comparable German population in which 30-45% of males and 45-55% of females age 18-29 years, and 45-70% of males and 55-85% of females age 30-59 years were non-smokers [22]. These significant gender-specific differences correspond to results from different surveys in the general German population [28] as well as the general European population [27], and correspond to previous studies on the health behaviour of medical students [25,29].

Medical education about the risks of smoking does not seem to be enough to lead to non-smoking behaviour. Health care professionals should be assisted in stopping smoking, for example by increasing the number of smoke-free health care facilities (e.g. smoke-free hospitals) [30].

### Consumption of illegal substances

The proportion of investigated physicians reporting consumption of illegal substances at least once is equal to the proportion in the general population reporting the same experience (5% versus less than 3% in the general population), and not lower [22]. In contrast, a French study showed that approximately 20% of general practitioners had smoked cannabis at least once [31]. The difference might derive from the fact that the majority of investigated physicians grew up in the German Democratic Republic, where illegal substances were not available or difficult to obtain [32].

On the other hand, the proportion of medical students reporting consumption of illegal substances was higher than in the general German population 18-29 years of age (15-25%) [22]. A Croatian study revealed similar results with 35% of medical students reporting consumption of illegal substances at least once [33]. The observed gender-specific differences in the group of medical students correspond to findings in the general German population: males reported to have consumed illegal substances three times more often than females [22]. Consequently, medical students require more information about the problems related to the consumption of illegal substances which can result in medical and psychiatric problems as well as legal problems. It can be assumed that most of the medical students do not know that a criminal record due to the consumption of illegal substances leads to rejection when applying for medical board registration.

## Conclusion

According to recent studies, the increase of work-related stress in health care professionals leads to an increase in unhealthy coping habits, such as alcohol consumption to relax, smoking to increase the number of breaks during working-hours or using drugs for stimulation or sedation [33-38]. Health care professionals should be supported in their position as role models for healthy lifestyle. Intensified education concerning healthy lifestyle and coping with consumption of alcohol, cigarettes and illegal substances during medical school could help improve these habits in health care professionals and their patients.

More studies are needed to investigate how early training concerning lifestyle improves health behaviour of health care professionals, and how this can optimise health consulting.

## Competing interests

The authors declare that they have no competing interests.

## Authors' contributions

AB conceived and designed the study. DM, AG and RV led the data collection. KV and AB analyzed and interpreted the data. ST assisted in data interpretation. KV drafted the manuscript. SRB, JK1 and JK2 critically reviewed the manuscript. All authors read and approved the final manuscript.

## Pre-publication history

The pre-publication history for this paper can be accessed here:

http://www.biomedcentral.com/1472-6963/9/219/prepub
